# A Mechanism Regulating the Onset of *Sox2* Expression in the Embryonic Neural Plate

**DOI:** 10.1371/journal.pbio.0060002

**Published:** 2008-01-08

**Authors:** Costis Papanayotou, Anne Mey, Anne-Marie Birot, Yasushi Saka, Sharon Boast, Jim C Smith, Jacques Samarut, Claudio D Stern

**Affiliations:** 1 Department of Anatomy & Developmental Biology, University College London, London, United Kingdom; 2 Institut de Génomique Fonctionnelle de Lyon, Université de Lyon, Lyon, France; 3 Ecole Normale Supérieure de Lyon, CNRS/INRA, Lyon, France; 4 Wellcome/Cancer Research UK Gurdon Institute for Cancer and Developmental Biology, Cambridge, United Kingdom; California Institute of Technology, United States of America

## Abstract

In vertebrate embryos, the earliest definitive marker for the neural plate, which will give rise to the entire central nervous system, is the transcription factor *Sox2*. Although some of the extracellular signals that regulate neural plate fate have been identified, we know very little about the mechanisms controlling *Sox2* expression and thus neural plate identity. Here, we use electroporation for gain- and loss-of-function in the chick embryo, in combination with bimolecular fluorescence complementation, two-hybrid screens, chromatin immunoprecipitation, and reporter assays to study protein interactions that regulate expression of N2, the earliest enhancer of *Sox2* to be activated and which directs expression to the largest part of the neural plate. We show that interactions between three coiled-coil domain proteins (ERNI, Geminin, and BERT), the heterochromatin proteins HP1α and HP1γ acting as repressors, and the chromatin-remodeling enzyme Brm acting as activator control the N2 enhancer. We propose that this mechanism regulates the timing of *Sox2* expression as part of the process of establishing neural plate identity.

## Author Summary

During early development, when the embryo has three layers of cells (ectoderm, mesoderm, and endoderm), a region of the ectoderm called the neural plate becomes specified to generate the entire nervous system. One of the earliest molecular markers for the neural plate is the transcription factor *Sox2*, which is critical for cells to acquire their neural fates and also defines neural progenitor character. We know very little about the intracellular mechanisms by which the neural plate cells acquire these fates. Here, we show that recruitment of transcriptional repressors to chromatin-remodeling complexes regulate the onset of *Sox2* expression. Competitive interactions between three proteins, ERNI, BERT, and Geminin, modulate the choice of repressors and regulate *Sox2* expression. During gastrulation, when the three embryonic cell layers form, ERNI recruits the repressor HP1γ to prevent Geminin from activating *Sox2* prematurely. By the end of gastrulation, this repression is counteracted by competitive binding of BERT to ERNI and Geminin, causing activation of *Sox2*. We propose that this mechanism regulates the timing of *Sox2* activation in the very early neural plate and thus helps to define the domain that will give rise to the nervous system.

## Abstract 3

The vertebrate nervous system develops from the neural plate, defined by the transcription factor *Sox2*. This paper uncovers a mechanism that regulates the timing of *Sox2* expression, involving interactions between several proteins and chromatin remodeling.

## Introduction

*Sox2* is a transcription factor that plays multiple critical roles during embryonic development in vertebrates. In embryonic stem (ES) cells, as well as in adult central nervous system (CNS) stem cells, *Sox2* expression is required for the maintenance of multipotency and for the ability of cells to self-renew [1]. *Sox2* is also expressed in cells that retain their ability to proliferate and/or acquire glial fates, whereas it is down-regulated in cells that become postmitotic and differentiate into neurons [2–4]. In addition, it is also transiently expressed outside the CNS in cranial sensory organs derived from the placodes and in subsets of peripheral nervous system (PNS) cells [5,6].

In all vertebrates studied to date, *Sox2* is also a general marker for the very early developing neural plate. In the chick, for example, *Sox2* expression starts at the late primitive streak stage (stages 4–4^+^ [7]) in the future neural territory [8,9]. A morphologically recognizable neural plate only becomes visible after the beginning of *Sox2* expression [8]. Importantly, Sox2 function is required for development of the neural plate [10]. Time-course experiments have shown that induction of *Sox2* requires the same period of exposure to organizer-derived signals (the tissue responsible for inducing the neural plate in the normal embryo [11–13]) as is required to induce a mature neural plate [14–17]. For these reasons, *Sox2* is considered to be the earliest definitive marker for the neural plate [18,19].

The complex expression profile of *Sox2* is controlled by multiple regulatory elements, each responsible for directing expression to a specific subset of expression sites. A very compelling analysis of the noncoding regions of *Sox2* in the chick embryo [20] revealed as many as 25 distinct conserved enhancers, of which two account for the expression of this gene in the early neural plate at stages 4^+^–5. One of these enhancers, named N2, is responsible for the initial expression (stage 4–4^+^) and is activated in a large domain corresponding to the entire forebrain/midbrain and most of the hindbrain. The other, N1, drives expression in the future caudal hindbrain and spinal cord and is activated a little later (around stage 5) [20,21]. To understand the processes that define the neural plate, it is essential to understand how the activity of these two elements, and especially N2, is regulated in the embryo.

Analysis of the N2 enhancer reveals multiple putative binding sites for known transcription factors [20,21]. However, the spatial and temporal expression patterns of these factors do not provide an obvious explanation for the time of onset of *Sox2* expression in normal development (unpublished data). Furthermore, to date, no single secreted factor or any combination thereof has been found to induce either *Sox2* expression or a neural plate in competent cells not normally fated to form part of the neural plate [13,19]. We therefore directed our attention to nuclear factors that might regulate this enhancer. Here, we provide evidence that a group of coiled-coil proteins interact with each other and with chromatin-remodeling factors and heterochromatin proteins to regulate the activity of the N2 enhancer. We propose that this is part of a mechanism that regulates the time of onset of expression of *Sox2* in the nascent neural plate.

## Results

### HP1α Inhibits *Sox2* Expression through a Brm-Dependent Mechanism

**Figure 1:**
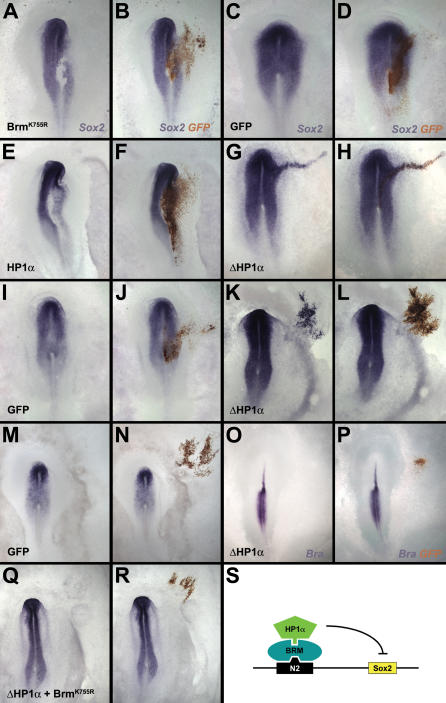
HP1α Inhibits *Sox2* (A, C, E, G, I, K, M, and Q) Embryos electroporated with *Brm^K755R^* (A), *GFP* (C), or *HP1α* (E) in the neural plate, *ΔHP1α* (G) or *GFP* (I) in the nonneural ectoderm, and *ΔHP1α* (K), *GFP* (M), and *ΔHP1α* together with *Brm^K755R^* (Q) in the extra-embryonic epiblast and stained for *Sox2* (purple). (B, D, F, H, J, L, N, and R) Subsequent staining for GFP (brown) marks the electroporated cells in the same embryos. (O and P) Embryo electroporated with *ΔHP1α* in the extra-embryonic epiblast and stained for *Brachyury* (purple) (O). In (P), the same embryo is stained for GFP (brown), which marks the electroporated cells. (S) These results suggest that HP1α bound to Brm on the N2 enhancer inhibits expression of *Sox2.* In this and subsequent figures, the construct electroporated is indicated on the lower left, and the probes used for in situ hybridization and antibody staining are on the lower right of the panels.

A recent study [22] using the P19 cell line demonstrated that the chromatin-remodeling enzyme Brahma (Brm) can activate *Sox2* by binding directly to the N2 enhancer. Is Brahma also involved in regulating *Sox2* expression in the normal embryo? To test this, we introduced a mutated version of Brahma (*Brm^K755R^*, which does not bind ATP and is therefore unable to remodel chromatin [23]) by electroporation into the prospective neural plate of embryos at stage 3–3^+^. This resulted in strong inhibition of *Sox2* expression in the electroporated domain (Figure 1A and 1B; 5/6), unlike controls electroporated with green fluorescent protein (GFP) (Figure 1C and 1D; 0/5 expressing).

**Figure 2:**
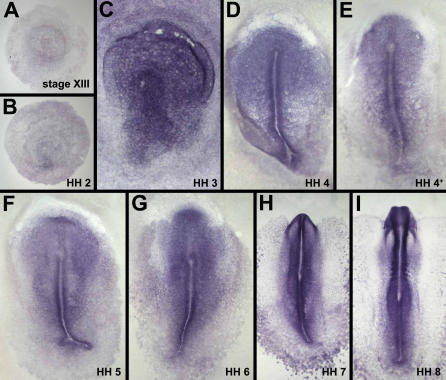
Expression of *HP1α* during Normal Development (A–C) Before and during gastrulation, *HP1α* is expressed throughout the embryo although its expression becomes gradually stronger in the prospective neural plate. (D–I) At the end of gastrulation (D), *HP1α* expression in the ectoderm becomes restricted in the prospective neural plate, where it gets stronger in subsequent stages (E–I) while it disappears from the nonneural ectoderm and the extra-embryonic epiblast. The number on each panel represents the embryonic stage according to Eyal-Giladi and Kochav [60] for pre-primitive streak stages (in Roman numerals), and Hamburger and Hamilton [7] for later stages (Arabic numerals).

However, *Brm* is expressed ubiquitously in the embryo [24]; what mechanisms prevent premature expression of *Sox2*? A good candidate is the transcriptional repressor HP1α, which binds directly to Brahma-related proteins at a highly conserved site [25] and which is also ubiquitously expressed in early embryos (Figure 2). Consistent with this, overexpression of HP1α in the neural plate represses *Sox2* (Figure 1E and 1F; 3/3). Could HP1α be an endogenous inhibitor of *Sox2* expression? To address this, we took advantage of the fact that both the chromoshadow domain and the chromodomain are necessary for the function of HP1 proteins [26,27]: targeting to chromatin requires interaction of the chromoshadow domain with a chromatin-tethered partner, as well as binding of the chromodomain to a methylated Lys^9^ of histone H3 [28]. We therefore made a dominant-negative form of HP1α (*ΔHP1α*) consisting of its isolated chromoshadow domain (which can bind to Brahma-related proteins but lacks repressor activity [25]). When *ΔHP1α* is misexpressed as a line extending from the neural plate into the peripheral, nonneural ectoderm (see Materials and Methods, “Design of assays”), *Sox2* is induced (Figure 1G and 1H; 6/7), whereas similar electroporation of GFP has no effect (Figure 1I and 1J; 0/8). This suggests that HP1α activity is required to prevent expression of *Sox2* in the nonneural ectoderm.

In embryos in which *ΔHP1α* was expressed as a line, we observed that *Sox2* was up-regulated, not only in the embryonic nonneural ectoderm (prospective epidermis), but also in the more peripheral area opaca epiblast (extraembryonic ectoderm) (Figure 1G). We were surprised by this observation because until now, various factors (such as bone morphogenetic protein [BMP] antagonists [16,17,19]) have been described that can expand the neural plate domain, but never as far as the extraembryonic epiblast, and none can induce a separate domain of *Sox2* expression in this region. The only treatment described to date that can induce neural markers in the area opaca is a graft of the organizer, Hensen's node, which is able to generate a complete, patterned nervous system in this region [13,29–32]. These observations define the area opaca as a particularly rigorous location in which to test for the neural inducing ability of factors (see Materials and Methods, “Design of assays”). Electroporation of *ΔHP1α* in this region dramatically induces *Sox2*, showing that HP1α normally represses *Sox2* expression (Figure 1K and 1L; 8/8). In contrast, electroporation of GFP in the same region has no effect (Figure 1M and 1N; 0/10). As an additional control, since HP1α may have more general activity as a transcriptional repressor, we also tested whether *ΔHP1α* can also induce other early embryonic genes using *Brachyury*, a marker for mesoderm expressed at this stage of development. It did not (Figure 1O and 1P; 0/3). This result also confirms that the induction of *Sox2* is direct, rather than a consequence of prior induction of mesoderm by ΔHP1α. Likewise, electroporation of *Brm^K755R^* or *Brm* had no effect on neural or mesoderm markers (0/5 for each; unpublished data). To test whether the inducing activity of ΔHP1α requires Brahma, we introduced *ΔHP1α* together with *Brm^K755R^*. This combination fails to induce *Sox2* (Figure 1Q and 1R; 0/12), suggesting that HP1α normally inhibits *Sox2* expression through a Brm-dependent mechanism (Figure 1S).

### Geminin Induces *Sox2* by a Brm-Dependent Mechanism

**Figure 3:**
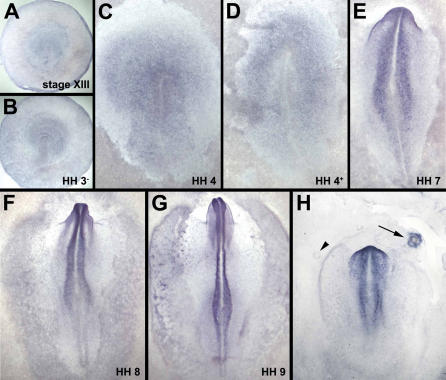
Expression of Chick *Geminin* during Normal Development and Its Regulation by FGF (A–G) *Geminin* is expressed in the embryonic epiblast from pre-primitive streak stages (A). As the embryo develops, its expression becomes restricted to the neural plate, where it intensifies (B–G). (H) An FGF-soaked bead up-regulates *Geminin* in the extra-embryonic epiblast (arrow); a control bead has been grafted on the contralateral side (arrowhead). Embryo stages as for Figure 2.

In *Xenopus*, the gene encoding the coiled-coil protein Geminin is expressed in the early prospective neural plate, and its misexpression induces neural markers [33]. More recently, it has been shown that Geminin interacts genetically with *Drosophila* Brahma, that it binds directly to its vertebrate homologs Brg1 and Brm (at the same site as does HP1α), and that Geminin knock-down abolishes *Sox2* expression [25,34]. Could Geminin be responsible for releasing the repression of Brm activity by HP1α? To test this, we cloned the chick homolog of *Geminin*. Before and during early gastrulation, *Geminin* is expressed in a large domain, which then (from stages 4–4^+^) becomes restricted to the neural plate (Figure 3). The early expression of chick *Geminin* resembles that of “pre-neural” genes (such as *Sox3*, *ERNI*, and *Churchill*), which precede the initiation of *Sox2* expression and which are induced by fibroblast growth factor 8 (FGF8) [8,9,35,36]. We therefore tested whether FGF can also induce *Geminin*. Indeed, *Geminin* can be induced by FGF8-soaked beads (9/10; Figure 3H, arrow), but not by control beads (0/10; Figure 3H, arrowhead).

**Figure 4:**
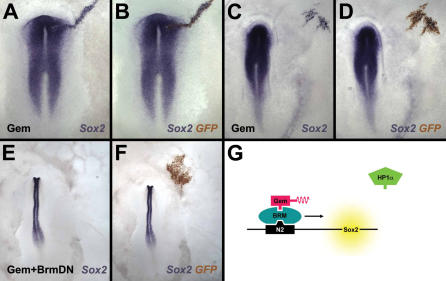
Geminin Induces *Sox2* (A–F) Embryos electroporated with *Geminin* (Gem) in the nonneural ectoderm (A) and *Geminin* (C) or *Geminin* together with *Brm^K755R^* (BrmDN) (E) in the extra-embryonic epiblast and stained for *Sox2* (purple). Subsequent staining for GFP (brown) marks the electroporated cells in the same embryos (B), (D), and (F). (G) We propose that Geminin displaces HP1α from its binding site on Brm, releasing its inhibition on *Sox2* expression.

When misexpressed as a line extending laterally from the neural plate, *Geminin* strongly induces ectopic *Sox2* (Figure 4A and 4B; 8/8). Geminin can also strongly induce *Sox2* expression when introduced into the extraembryonic epiblast (Figure 4C and 4D; 20/20). To test whether this induction requires the chromatin-remodeling activity of Brm, we cointroduced *Geminin* and *Brm^K755R^*: the mutated chromatin remodeler abolishes the induction of *Sox2* by Geminin (Figure 4E and 4F; 0/15), suggesting that Brm activity is required for *Sox2* induction by Geminin. Together, these results suggest that early in development *Sox2* expression is constitutively repressed by the presence of HP1α bound to Brm at the N2 enhancer of *Sox2*, and that later in development, FGF may release this inhibition through induction of Geminin, which competes HP1α away from the protein complex (Figure 4G).

### ERNI Inhibits *Sox2* Induction by Geminin

**Figure 5:**
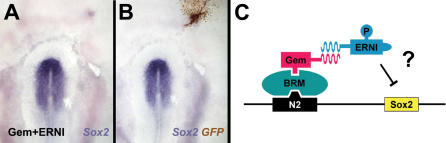
ERNI Blocks Induction of *Sox2* by Geminin (A and B) Embryo electroporated with *Geminin* and *ERNI* in the extraembryonic epiblast and stained for *Sox2* (A) and GFP (B) to mark the electroporated cells. (C) We propose that ERNI inhibits the induction of *Sox2* by Geminin.

*Geminin* is already expressed at the beginning of gastrulation (Figure 3), long before *Sox2* (which appears at stage 4 [8,9]), suggesting that an additional mechanism must exist to prevent premature *Sox2* activation. A good candidate for this repression is *ERNI*, which is broadly expressed in the epiblast at early stages but is rapidly down-regulated from the prospective neural plate at stage 4^+^ [36], around the time when *Sox2* starts to be expressed. To test whether ERNI can inhibit the induction of *Sox2* by Geminin, we cointroduced them into the area opaca: *ERNI* does indeed inhibit the induction of *Sox2* by Geminin (6/14 with very weak induction, 8/14 with no induction; Figure 5A–5C).

### Mechanism of Repression by ERNI

#### ERNI and Geminin bind through their coiled-coil domains.

By what mechanism does ERNI exert its inhibition? ERNI contains two putative functional domains: a coiled-coil domain in its N-terminal half (amino acids [aa] 79–137) and a likely phosphorylation site (aa 222–228) [36]. Since coiled-coil domains are often involved in protein–protein interactions, and since both ERNI and Geminin contain such a domain, we tested the possibility that ERNI can bind to Geminin and/or to itself. As no antibodies to ERNI or chick Geminin are available, precluding coimmunoprecipitation assays, we used bimolecular fluorescence complementation (BiFCo), a powerful technique allowing protein interactions to be visualized within living cells [37,38]. All possible combinations of ERNI and Geminin fusions to the N- and C-termini of the yellow fluorescent protein (YFP) variant Venus [39] were transfected pairwise into COS cells so that whenever an interaction occurs, fluorescence is seen. This revealed that ERNI and Geminin can associate as homo- or heterodimers, unlike several controls, including other coiled-coil proteins (Table 1). The same results were obtained in vivo when the constructs are electroporated into early chick embryos (unpublished data). To test whether this interaction occurs through the coiled-coil domain, we repeated this experiment using the isolated coiled-coil domains of ERNI and Geminin. This revealed that the isolated coiled-coil domains of ERNI and Geminin are sufficient for their homo- and heterodimerization (Table 1).

**Table 1:**
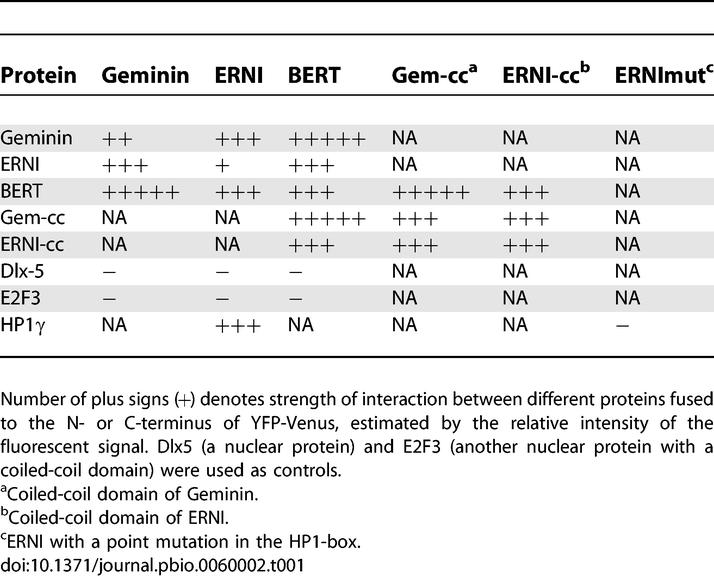
Protein–Protein Interactions Confirmed by BiFCo

#### The C-terminus of ERNI is important for its function.

**Figure 6:**
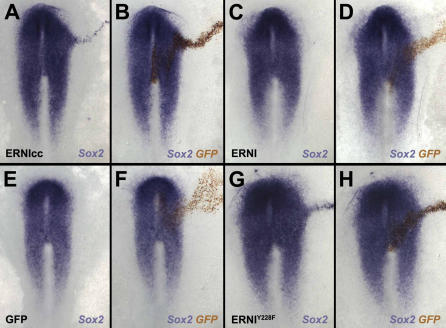
Mutated Forms of ERNI Induce *Sox2* (A, C, E, and G) Embryos electroporated with the isolated coiled-coil domain of ERNI (*ERNIcc*) (A), *ERNI* (C), *GFP* (E), or *ERNI^Y228F^* (G) in the nonneural ectoderm and stained for *Sox2* (purple). (B, D, F, and H) Subsequent staining for GFP (brown) marks the electroporated cells in the same embryos.

Since ERNI lacks any recognizable repression motif, it is possible that it antagonizes the activity of Geminin by recruiting other proteins that interact with regions outside the coiled-coil domain (which is involved in binding to Geminin) (Figure 5C). If so, constructs lacking regions outside the coiled-coil domain should still bind to Geminin but fail to recruit the repressor(s), therefore acting as dominant-negative forms. To test this, we misexpressed the isolated coiled-coil (*ERNIcc*) by electroporation in late primitive streak stage chick embryos. This induces strong expression of *Sox2* (Figure 6A and 6B; 12/12), unlike full-length *ERNI* (Figure 6C and 6D; 0/8) or *GFP* alone (Figure 6E and 6F; 0/8). *ERNIcc* did not induce expression of other markers (*Brachyury* (0/6), *Chordin* (0/4), or *BMP4* (0/5); unpublished data), confirming that the induction of *Sox2* by *ERNIcc* is direct and specific. Misexpression of *ERNI* containing a point mutation in the putative phosphorylation site (*ERNI^Y228F^*) has the same effect as misexpression of the isolated coiled-coil domain (Figure 6G and 6H; 9/9), suggesting that ERNI phosphorylation is important for its function.

#### HP1γ binds to the C-terminus of ERNI and is required for repression of Sox2.

**Figure 7:**
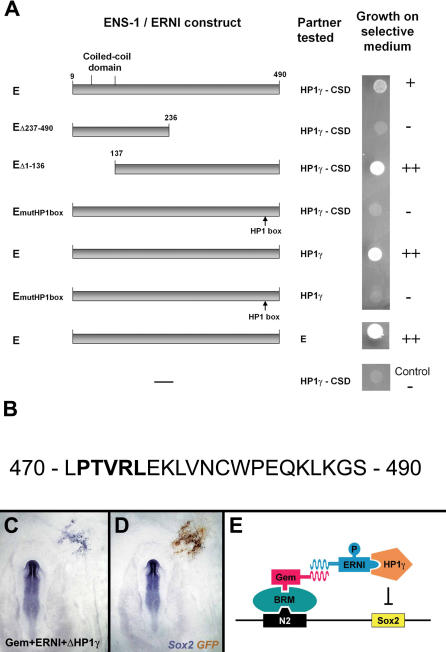
ERNI Inhibits *Sox2* through HP1γ (A) A two-hybrid screen reveals that HP1γ interacts with the C-terminus of ERNI: ++, +, or − indicates growth induced by interaction of ENS-1/ERNI (E) with HP1γ/CHCB2 or with itself. Partially deleted or HP1-box–mutated forms are indicated. HP1γ-CSD: carboxy-terminal 87 aa of HP1γ containing the chromoshadow domain (CSD). (B) Partial amino acid sequence of ENS-1/ERNI: the pentapeptide PXVXL, necessary for specific interaction with the CSD of HP1γ/CHCB2, is in bold. (C) Embryo electroporated with *Geminin*, *ERNI*, and *ΔHP1γ* in the extra-embryonic epiblast (G) and stained for *Sox2* (purple). (D) The same embryo after staining for GFP (brown) to mark electroporated cells. (E) We propose that ERNI inhibits *Sox2* expression by recruiting HP1γ to the *N2* enhancer.

The above results predict that ERNI might inhibit *Sox2* by recruiting one or more inhibitory proteins to the complex (Figure 5C). To identify such an inhibitor, expressed at very early stages of development, we took advantage of the fact that *ERNI* is also strongly expressed in undifferentiated chicken embryonic stem (cES) cells (where it was identified as *ENS-1* [40]). We therefore undertook a two-hybrid screen using *ENS-1* as bait and a cDNA library from undifferentiated cES cells. This identified, among several candidates, *CHCB2*, the chick homolog of *HP1γ*, a chromatin modifier with repressor activity [41] (Figure 7A). HP1γ is related to HP1α but does not bind directly to Brahma-related proteins [25]. Further two-hybrid assays using an *ERNI/ENS-1* clone confirmed (see above and Table 1) that ERNI can form homodimers and that this interaction requires the coiled-coil domain. In contrast, interaction with HP1γ is mediated by a short sequence (HP1-box, PxVxL [28]) close to the C-terminus of ERNI (Figure 7A and 7B). The related protein HP1β does not bind significantly to ERNI in the same assay (unpublished data). HP1γ interacts with ERNI through the chromoshadow domain [42,43] of the former (unpublished data). To confirm these interactions by an independent method, we used BiFCo in cES cells, which showed that HP1γ can bind to full-length ERNI, but not to ERNI with a mutated HP1-box (Table 1). We also used this method to test whether ERNI can bind to HP1α: no significant binding was seen (unpublished data). These findings suggest that ERNI inhibits *Sox2* expression by binding to Geminin through their respective coiled-coil domains and by specific recruitment of the HP1γ repressor through a C-terminal HP1-box.

These findings predict that a dominant-negative form of HP1γ (comprising the chromoshadow domain but lacking the chromodomain, which is required for repression [26–28]) should be able to relieve the inhibition by ERNI of *Sox2* induction by Geminin (see above and Figure 5A–5C). To test this, we coelectroporated *Geminin* and *ERNI* together with the isolated chromoshadow domain of *HP1γ* (*ΔHP1γ*) into the area opaca. This combination of factors induces *Sox2* (Figure 7C and 7D; 7/8), suggesting that HP1γ is indeed required for the inhibitory activity of ERNI (Figure 7E).

**Figure 8:**
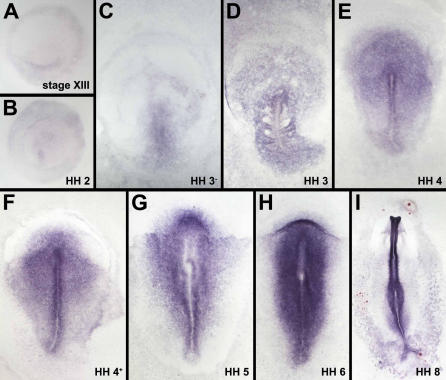
Expression of *HP1γ* during Normal Development (A) Before primitive streak formation, *HP1γ* is expressed in the extra-embryonic epiblast. (B–D) When the primitive streak forms, *HP1γ* expression appears in the embryonic epiblast where it gradually becomes stronger in the prospective neural plate. (E–I) At the end of gastrulation (E), its expression in the ectoderm becomes restricted in the prospective neural plate, where it gets stronger at subsequent stages (F–I), while it disappears from the nonneural ectoderm and extra-embryonic epiblast.

Is HP1γ expressed at the right time and place to play a role in regulating the onset of *Sox2* expression? In situ hybridization reveals low-level, ubiquitous expression from early primitive streak stages, increasing in the prospective neural epiblast from stage 3^+^–4 (Figure 8).

### BERT, an Endogenous ERNI Antagonist

**Figure 9:**
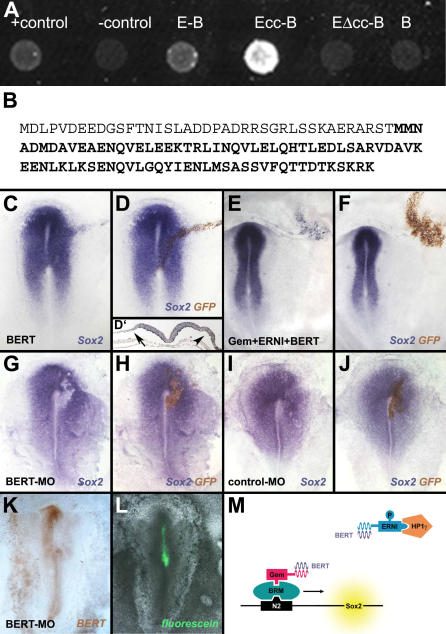
BERT Releases Geminin from the Inhibition of ERNI (A) Representative colonies from the two-hybrid screen (left to right): positive (+) control, negative (−) control, ERNI+BERT (E-B), ERNIcc+BERT (Ecc-B), ERNI without coiled-coil domain + BERT (EΔcc-B), and BERT alone (B). (B) Amino acid sequence of BERT. The sequence originally isolated is shown in bold; the rest encodes a putative upstream exon. (C–F) Embryos electroporated with BERT (C) in the nonneural ectoderm or Geminin, ERNI, and BERT (E) in the extra-embryonic epiblast and stained for *Sox2* (purple). The same embryos after staining for GFP (brown) to mark electroporated cells (D) and (F). A section of the embryo in (D) reveals that the induced epiblast acquires a neural plate–like morphology (D′). (G–J) Embryos electroporated with a MO against BERT (G) or a control MO (I) in the prospective neural plate and stained for *Sox2* (purple). In (H) and (J), the same embryos are stained with anti-fluorescein to detect the MO (brown). (K and L) Embryo electroporated with the MO against BERT in the neural plate and immunostained for BERT protein (brown, [K]). (L) The same embryo under fluorescence to show the cells electroporated with the fluorescein-labeled MO. (M) We propose that BERT disrupts the interaction between Geminin and ERNI, displacing HP1γ from the *N2* enhancer and thus allowing Geminin/Brahma to induce *Sox2* expression.

**Figure 10:**
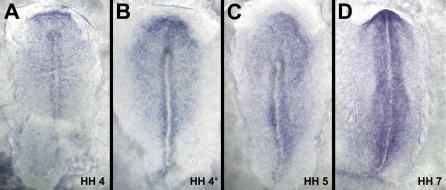
Expression of *BERT* during Normal Development BERT is expressed ubiquitously in the chick embryo albeit at low levels. At the end of gastrulation (A), its expression is up-regulated in the prospective neural plate, where it becomes stronger during subsequent stages (B–D).

The above findings are consistent with the idea that ERNI normally functions to repress *Sox2* expression at very early stages of development. However, it is unlikely that down-regulation of *ERNI* transcription is sufficient to relieve this inhibition because *Sox2* expression begins at stages 4–4^+^ [7], when some *ERNI* transcripts can still be detected within the prospective neural plate [36]. Therefore, an endogenous inhibitor is likely to exist whose expression should begin at around this time (stage 4–4^+^). To identify such an inhibitor, a two-hybrid screen was performed using ERNI as bait and a library of cDNAs from stage 3–6 chick embryos (Figure 9A). Only one partner was found, encoding a small coiled-coil domain protein which we named BERT (Figure 9B). An equivalent human protein (SCOCO, corresponding to the fragment shown in bold in Figure 9B) was previously isolated as a partner of human ARL1, a component of the Golgi apparatus [44], and a nematode homolog (*unc69*) was found to be essential for neural development [45]. *BERT* is expressed ubiquitously at low levels at all stages, but is up-regulated specifically in the prospective neural plate from stage 4–4^+^ (Figure 10), just prior to when *Sox2* expression appears [8,9]. When misexpressed as a line across the nonneural epiblast, *BERT* acts like the dominant-negative *ERNI* constructs: it induces strong expression of *Sox2* (Figure 9C and 9D; 15/15), which also acquires a neural plate-like morphology (Figure 9D′: note that the thickened ectoderm characteristic of the neural plate has greatly expanded on the electroporated side; see arrowhead on right). Mesodermal markers (*Brachyury*, *Chordin*, and *BMP4*; 0/5, 0/4, 0/4, respectively; unpublished data) are not induced, showing that this expansion of the neural plate is a direct effect. These findings indicate that BERT has an activity compatible with it being an endogenous antagonist of ERNI, regulating not only *Sox2* expression, but also the onset of neural plate development. To confirm this, we examined the effects of coexpressing BERT with Geminin+ERNI (which does not induce *Sox2*; see above and Figure 5A and 5B) in the area opaca. Indeed, when all three constructs are cointroduced, induction of *Sox2* is seen (12/12; Figure 9E and 9F).

Is BERT required to control the onset of *Sox2* expression in the neural plate? To address this, we designed a fluorescein-labeled Morpholino oligonucleotide (MO) to the 5′ end of the coding sequence (see Materials and Methods) and introduced this (together with GFP) by electroporation into the prospective neural plate at stage 3–3^+^ and examined *Sox2* expression at stages 4^+^–5. BERT-MO caused down-regulation of *Sox2* expression in this domain (Figure 9G and 9H; 5/6), unlike control MO (Figure 9I and 9J; 0/5). Staining of BERT-MO–electroporated embryos with an antibody against BERT/SCOCO (see Materials and Methods) confirmed that the MO does indeed inhibit translation of BERT protein (Figure 9K) in the electroporated domain (Figure 9L). Together, these findings implicate BERT as an endogenous antagonist of ERNI, required to regulate the onset of *Sox2* expression in the neural plate.

**Figure 11:**
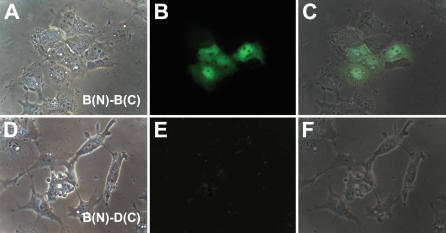
Direct Interactions between the Coiled-Coil Proteins (A–C) Example of a positive interaction between BERT and itself (homodimerization). B(C): BERT-Venus(C); B(N), BERT-Venus(N). (D and F) Example of a negative control, BERT, and another coiled-coil protein, E2F3. D(C), E2F5-Venus(C). (A) and (D) Phase contrast, (B) and (E) fluorescence, (C) and (F) merged phase contrast and fluorescence images.

**Figure 12:**
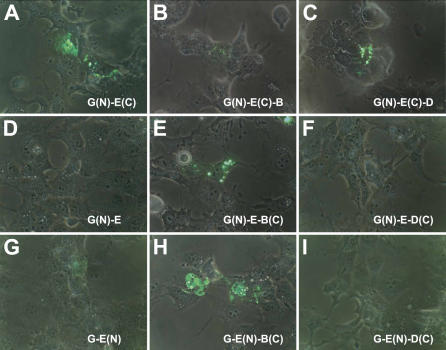
BiFCo Competition Assays (A and B) BERT disrupts Geminin-Venus(N)::ERNI-Venus(C) heterodimers (G(N)-E(C)). B, BERT. (D and E) BERT-Venus(C) disrupts Geminin-Venus(N)::ERNI heterodimers through its association with Geminin-Venus(N). B(C), Bert-Venus(C); E, ERNI; G(N), Geminin-Venus(N). (G and H) BERT-Venus(C) disrupts Geminin::ERNI-Venus(N) heterodimers through its association with ERNI-Venus(N). B(C), BERT-Venus(C); E(N), ERNI-Venus(N); G, Geminin. (C), (F), and (I) are the respective controls using the noninteracting Dlx5 protein as competitor. D(C), Dlx5-Venus(C)

From the results presented above, the evidence that BERT binds to ERNI directly is based entirely on the two-hybrid screen used to isolate BERT. To confirm that the two proteins can interact physically, we used BiFCo assays, which further revealed that BERT, Geminin, and ERNI all bind to each other through their coiled-coil domains (Figure 11 and Table 1). This finding raises the possibility that BERT disrupts Geminin-ERNI dimers by binding to both proteins, thus removing ERNI-HP1γ from the complex to activate *Sox2* (Figure 9M). To test this further, we used BiFCo competition assays (Figure 12). When BERT is added to Geminin-Venus(N)+ERNI-Venus(C), fluorescence is lost (Figure 12A and 12B). When Dlx5 is used as a control instead of BERT in this assay, there is no effect (Figure 12C). Conversely, when BERT-Venus(C) is added to Geminin-Venus(N)+ERNI, fluorescence is generated (Figure 12D and 12E), and this is not mimicked by addition of Dlx5-Venus(C) (Figure 12F). Likewise, when BERT-Venus(C) is added to Geminin+ERNI-Venus(N), fluorescence is produced (Figure 12G and 12H), which is not mimicked by the use of Dlx5-Venus(C) instead of BERT-Venus(C) (Figure 12I). Together, these findings support the idea that BERT can disrupt Geminin-ERNI heterodimers by binding to both proteins.

### The Protein Complex Regulates the Activity of the N2 Enhancer of *Sox2*

**Figure 13:**
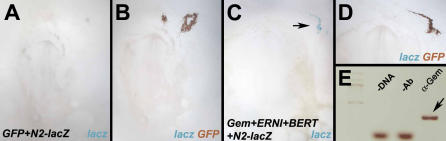
Interaction of Geminin, ERNI, and BERT on the N2 Enhancer (A–D) Electroporation of GFP with *N2-TK-LacZ* does not activate the reporter (A), whereas electroporation of Geminin+ERNI+BERT with *N2-TK-LacZ* does ([C]; LacZ in blue). In (B) and (D), the same embryos are stained for GFP to mark the electroporated cells. (E) ChIP assay on chromatin from E7.5 mouse neural plate, demonstrating a direct interaction between Geminin and the N2 enhancer. ab, antibody.

The experiments described above tested the protein–protein interactions and their effects on *Sox2* expression, but their physical association with the N2 enhancer was extrapolated from published results in a cultured cell line, unrelated to the early neural plate [22]. To test whether these interactions can regulate *Sox2* expression directly at the N2 enhancer, we coelectroporated a reporter construct consisting of the N2 enhancer and a minimal TK promoter [20] driving expression of LacZ together with either *Geminin* alone, with *Geminin*+*ERNI*, or with *Geminin*+*ERNI*+*BERT*, into the extraembryonic epiblast. No expression of the reporter was seen when it was coelectroporated with the control construct *pCAβ-GFP* (Figure 13A and 13B; 0/16) or with *Geminin*+*ERNI* (unpublished data; 0/6). However, expression was induced by both Geminin (unpublished data; 6/6) and by Geminin+ERNI+BERT (Figure 13C and 13D; 5/5). This shows that ERNI can block the activity of the N2 enhancer of *Sox2* and that BERT inhibits this.

Finally, to confirm that these proteins do indeed interact physically with the N2 enhancer, we conducted chromatin immunoprecipitation (ChIP) assays using chromatin extracted from embryonic day (E)7.5 mouse embryos and an antibody against mouse Geminin. The antibody specifically precipitates the N2 enhancer of *Sox2* (Figure 13E, lane 3), unlike control experiments performed either without chromatin (Figure 13E, lane 1) or without anti-Geminin antibody (Figure 13E, lane 2). These findings demonstrate that Geminin does indeed associate physically with the N2 enhancer of *Sox2* in vivo at an appropriate stage in development.

## Discussion

*Sox2* is an important gene that plays multiple roles in development especially in controlling cell fate and proliferation. Its expression pattern is complex and regulated by multiple noncoding elements [20,21]. In the normal embryo, one of the earliest conserved sites of expression is the nascent neural plate, where *Sox2* constitutes the earliest definitive marker for this tissue. It is therefore of particular interest to understand the mechanisms that regulate the location and timing of expression of this gene in the neural plate, as this process is critical for normal nervous system development. Here, we propose that interactions between several coiled-coil proteins, heterochromatin proteins, and chromatin-remodeling molecules regulate the time of onset of *Sox2* expression in the chick neural plate.

### A Simple Model

The most parsimonious model to explain our findings in terms of how *Sox2* expression is regulated in the early neural plate comprises the four steps shown in Figures 1S, 4G, 7E, and 9M. Since *Brm* and *HP1α* are expressed ubiquitously ([24] and results from the present study), we propose that there is a basal state in which Brm is bound to the N2 enhancer of *Sox2* [22], but the latter is not expressed because the repressor HP1α occupies the chromoshadow-binding domain of Brm (Figure 1S). Early in development, FGF activity induces both *ERNI* [18,36] and *Geminin* (this study) in the epiblast. Geminin binds to the chromoshadow-binding domain of Brm, displacing HP1α (Figure 4G). However, the interaction of ERNI with Geminin recruits the transcriptional repressor HP1γ, thus continuing to prevent premature expression of *Sox2* in the epiblast (Figure 7E). Later in development (stage 4–4^+^), BERT is up-regulated within the neural plate, where it binds to both ERNI and Geminin and displaces ERNI-HP1γ complexes away from Brm, freeing the latter to activate N2 and thus *Sox2* expression (Figure 9M). At around the same time, *ERNI* transcription starts to be down-regulated in the neural plate. This model accommodates all of our results and those in the literature; its significance is explored further in the following sections.

### Molecular Interactions Regulating the Timing of Gene Expression

The N2 enhancer of Sox2 is about 550 bp long and is predicted to contain multiple binding sites for transcription factors [20], many of which are expressed in the epiblast prior to the stage at which *Sox2* expression is initiated. In principle, binding of the appropriate activators to the N2 enhancer should turn on *Sox2*. However, the spatial and temporal patterns of expression of these factors do not account for the timing or spatial distribution of *Sox2* transcription at this stage in development, as many of them are expressed ubiquitously (unpublished data). We therefore propose that, irrespective of the binding of putative activators to the N2 enhancer, the conformation of chromatin, maintained in a closed configuration by HP1 proteins, prevents activation at early stages. It is only when HP1 proteins are removed and the chromatin-remodeling activity of Brm is released that N2 is activated.

Chromatin-remodeling complexes may turn out to have a widespread role in the transcriptional activation of specific genes, as exemplified by Smad-activated genes whose transcriptional regulation also requires the activity of such complexes [46]. Likewise during skeletal muscle differentiation, chromatin-binding proteins “mark the spot” for activation of genes by other transcription factors together with chromatin remodeling by SWI/SNF proteins: MyoD binding to chromatin is regulated by the homeodomain protein Pbx1 in cooperation with the Brahma-related enzyme Brg1 [47–50]. To our knowledge, however, this is the first report suggesting that a SWI/SNF chromatin-remodeling complex can recruit HP1 proteins to a specific enhancer to repress transcription of a target gene.

### Mechanisms Regulating the Timing of Neural Plate Formation

Our model proposes mutually inhibitory interactions between several proteins. Why does *Sox2* need to be regulated by such a complex mechanism, rather than by merely recruiting a single or a few activators to a simple enhancer? We suggest that this is one in a series of steps that act to separate different functions for signals that are common to different developmental processes.

Previously, we showed that 3–5 h of exposure to signals from the organizer (Hensen's node) is sufficient to induce transient expression of the pre-neural marker *Sox3*, but not sufficient to induce later neural plate markers (such as *Sox2*), and that the BMP antagonist Chordin can stabilize the expression of *Sox3* induced by such a graft (but again not induce *Sox2*) [16]. Based on these findings, we conducted a screen to identify genes induced within 5 h of exposure to the organizer [36]. We identified several genes induced within this time, among them *ERNI*, which is induced very rapidly, within 1–2 h. FGF8 is sufficient to mimic this effect, and during normal development, *ERNI* is expressed even before gastrulation, in a domain identical to that covered by the underlying hypoblast (which expresses *FGF8*).

FGF is required for both mesodermal [51–54] and neural induction [36,55,56]. How do cells that have received FGF signals decide between these two incompatible fates? A likely scenario is that cooperation with other factors, present at different times and in different locations, contributes to refine this choice. To allow this to happen, it may be necessary for cells to retain a “memory” that they have received FGF signals yet be prevented from being allocated prematurely to inappropriate fates. ERNI appears to fulfill such a role: while it is expressed, cells are multipotent, as its early domain of expression encompasses the prospective neural and mesendodermal domains as well as some nonneural ectoderm. At the end of gastrulation, *ERNI* transcription starts to be down-regulated from the future neural plate, remaining only at the border between neural and epidermal domains [36,57]. At the same time, *BERT* is up-regulated in the domain that is losing *ERNI* expression while *Sox2* starts to be expressed in the same domain (stage 4–4^+^). This sequence of events could help to explain why it takes such a long time (about 9 h) following a graft of a node for *Sox2* expression to begin and for a neural plate to be induced [14–17]. Consistent with the proposal that ERNI is part of a mechanism to prevent premature expression of *Sox2*, we have observed that transfection of *BERT* into the prospective neural plate region of stage 2–3 embryos can induce premature expression of *Sox2* (unpublished data).

The present and previous studies [36] reveal that FGF signaling activates *ERNI* as well as *Sox3* and *Geminin* expression in the epiblast. However, FGF does not induce *BERT*, whose expression is also not regulated by BMP antagonists or any combination of known factors implicated in neural induction to date (unpublished data). In future, it will be interesting to determine whether BERT is induced by some other combination of factors or whether its expression is regulated simply by a cell-autonomous timer in cells that are still in the epiblast at the end of gastrulation, but does not require input from other cells.

In all likelihood, the mechanisms responsible for regulating *Sox2* expression and the acquisition of neural fate will turn out to be considerably more complex, and our model does not rule out additional mechanisms. It will be interesting in future to investigate whether other developmentally expressed genes are regulated by similar processes.

### Conclusions

Our findings provide a mechanism for how *Sox2* expression is initiated as part of the events that define the early neural plate. We propose that ERNI functions as an inhibitor of premature *Sox2* expression during early gastrulation: cells expressing *ERNI* are multipotent and can generate any cell type. Cells that remain in the epiblast at the end of gastrulation and acquire expression of *BERT* to activate *Sox2*, which, most likely together with other genes involved in neural specification, assigns a neural plate fate.

## Materials and Methods

### Chick experiments.

Fertile hens' eggs (Brown Bovan Gold; Henry Stewart & Co.) were incubated at 38 °C to the desired stages. Electroporations were performed as described [35]. The coding region of full-length ERNI, ERNI coiled-coil domain (aa 1–164), chick BERT, chick Geminin, human Brm^K755R^ (kind gift from Dr A Imbalzano), mouse HP1α, mouse HP1α chromoshadow domain (aa 106–180), and mouse HP1γ chromoshadow domain (aa 118–176) were cloned into *pCAβ* and electroporated at 0.2 μg/μl (except ERNI and Brm^K755R^ and HP1α, which were used at 0.4 μg/μl) together with 1 μg/μl of *pCAβ*-*GFP*, which was used to mark the electroporated cells. The *N2-TK-LacZ* reporter plasmid was constructed from *N2-TK-GFP*, kindly provided by Dr H. Kondoh, and was electroporated at 1 μg/μl. FGF8b (Sigma) was delivered bound to heparin beads (prepared as described [19]) at 50 μg/ml. In situ hybridization and immunostaining for GFP were performed as described [35].

### Design of assays.

To establish the role of different components in regulating the expression of *Sox2*, three different types of assays were used for gain- and loss-of-function experiments. First, to assess the effects on endogenous expression of *Sox2* in the normal neural plate, constructs were introduced into the prospective neural plate at mid-primitive streak stage (stage 3–3^+^) and the embryos incubated about 6–9 h so that the embryo had reached stages 4^+^–7, just beyond the stages at which *Sox2* expression begins (4^+^) and also because at these stages the neural plate is still open, allowing easier visualization of expanded expression. Please note that stages 4^+^–7 are particularly short, this entire period lasting only about 3 ± 1.5 h at 38 °C. To determine whether a construct can induce ectopic expression of *Sox2*, two different locations were chosen. In one set of assays, the construct is introduced as a continuous line between the prospective neural plate of the embryo and the inner aspect of the extraembryonic epiblast, covering most of the prospective epidermis. In the other assay, the construct is introduced as a discrete domain within the inner third of the extraembryonic (area opaca) epiblast and the embryos incubated 12–15 h (by which time they have reached stages 6–9).

The reasons for choosing both of the latter two assays for induction is that extensive embryological studies have revealed differences in their reactivity to neural inducing stimuli. For example, inhibition of BMP signaling is sufficient to expand the endogenous neural plate laterally (and BMP misexpression to narrow it), but only when the territory is continuous with the embryo's own neural plate [13,16,17], suggesting that induction of neural markers by certain stimuli in this region requires cellular continuity with the neural plate and/or its border. On the other hand, a graft of the organizer (Hensen's node) is able to induce a complete, patterned ectopic nervous system from the extraembryonic epiblast of the inner area opaca [13,29–32]. A period of 9–13-h contact is required to induce *Sox2* after a graft of the organizer, which is why 12–15 h was chosen in this assay. To date, no single factor or any combination thereof has been found to mimic this activity of the organizer. It is therefore particularly important, to assess the full inducing properties of a treatment, to test its ability to induce *Sox2* in the area opaca. We therefore used all three assays to compile a more comprehensive understanding of the inducing or inhibiting activities of each of the constructs in this study.

### Morpholino experiments.

A translation-blocking MO against BERT with the sequence CAGCGTCCATGTCAGCGTTCATCAT, targeting the 5′ end of the ORF of the gene or a standard control MO (Gene Tools LLC), both labeled with fluorescein, were electroporated by injecting a small volume (about 0.1 μl) of a stock of the MO at 1 mM exactly as described for electroporation of constructs (see above). Antibody against human SCOCO was kindly provided by Dr. Richard Kahn. This was used in whole mounts by indirect immunoperoxidase with anti-rabbit-HRP using the same method as described for GFP (see above).

### Two-hybrid screens.

For two-hybrid screens with embryonic cDNA, poly-A RNA was isolated from 600 chick embryos (stage 3–6) using the Ambion Poly(A)Pure Kit. The mRNA was used to synthesize a cDNA library which was cloned into the *pMyr* vector using the CytoTrap XR Library Construction Kit (Stratagene). The library was transformed into XL10-Gold Ultracompetent Cells (Stratagene). Full-length *ERNI* was cloned into the *pSOS* vector and used as bait in the CytoTrap two-hybrid screen, which was performed according to the manufacturer's instructions (Stratagene).

For two-hybrid screens on chick ES cells, poly-A RNA was isolated from ES cells [58]. cDNA was synthesized using Stratagene's cDNA synthesis kit and introduced into *pGAD424* vector (Clontech), and this was transformed into XL1-blue MRF' bacteria by electroporation. All plasmids, yeast strains, and media used were purchased from Clontech. The bait *ENS-1/ERNI* coding sequence was cloned in NdeI/SalI sites of *pGBKT7*, introduced into AH109 yeast, and checked for lack of self-activation of the reporter. Screening was performed according to the Yeast Protocols Handbook (Clontech). *pGAD424* recombinant plasmids from 18 candidates were purified, of which seven encoded the CHCB2 protein [41] and all included the chromoshadow domain. The smallest one, encoding the 87 carboxy-terminal amino acids, was used in further experiments. The full *ENS-1/ERNI* coding sequence was cloned into *pGADT7* and various truncated forms (Figure 5) subcloned into *pGBKT7*. Point mutations were introduced into *pGBKT7:ENS-1* using the QuikChange site-directed mutagenesis kit from Stratagene and checked by sequencing. Yeast two-hybrid assays were performed by rapid cotransformation of strain AH109.

### Cloning of chick Geminin.

The *Xenopus* Geminin amino acid sequence was used to BLAST the GenBank EST database. The full-length chick homolog sequence was recovered and cloned by PCR from the CytoTrap cDNA library described above.

### BiFCo experiments.

The N- and C-terminal halves of Venus (aa 1–154 and 155–229) were PCR-amplified from pCS2 vectors and cloned into pcDNA3.1A. *Geminin*, *ERNI*, *BERT*, and human E2F3 were cloned in frame into the 5′ end of each of the two Venus halves, giving rise to six plasmids expressing each of the three genes fused to either of the two Venus halves. Dlx5 control vectors were a kind gift of Andrew Bailey. COS cells and cES cells were transfected as described [16], and the cells were observed the next day by epifluorescence in a compound microscope.

### ChIP assay.

The method used closely followed one previously described [59]. Briefly, 20 E7.5 mouse embryos were fixed in 4% formaldehyde, homogenized in lysis buffer, and sonicated. Cell extracts were harvested by centrifugation, incubated overnight with an antibody against mouse Geminin (Santa Cruz Biotechnology FL-209, 5 μg), and then immunoprecipitated with Protein-A-Sepharose. Precipitates were heated to reverse the formaldehyde cross-linking. The DNA fragments in the precipitates were purified by phenol/chloroform extraction and EtOH precipitation and used as a template for a PCR, using the following mouse N2-specific primers: forward: AACTCTCATAGCCCTAACTGTC, reverse: CCCTCCTCTCCTAATCTCCTTATGG. After 20 cycles of amplification, one-tenth of the reaction product was used as a template for a second round of a further 20 cycles. The final PCR products were run on a 1% agarose gel.

## Supporting Information

### Accession Numbers

The GenBank (http://www.ncbi.nlm.nih.gov/Genbank) accession number for the chick homolog of *Geminin* is EU118174, and for BERT, it is EU118175.

## Abbreviations

aa: amino acid
BiFCo: bimolecular fluorescence complementation
BMP: bone morphogenetic protein
cES: chicken embryonic stem
ES: embryonic stem
FGF: fibroblast growth factor
GFP: green fluorescent protein
MO: Morpholino oligonucleotide
